# A low-potential terminal oxidase associated with the iron-only nitrogenase from the nitrogen-fixing bacterium *Azotobacter vinelandii*

**DOI:** 10.1074/jbc.RA118.007285

**Published:** 2019-05-01

**Authors:** Febin Varghese, Burak Veli Kabasakal, Charles A. R. Cotton, Jörg Schumacher, A. William Rutherford, Andrea Fantuzzi, James W. Murray

**Affiliations:** From the Department of Life Sciences, Imperial College London, London SW7 2AZ, United Kingdom

**Keywords:** nitrogen fixation, nitrogenase, dioxygenase, enzyme structure, oxidase

## Abstract

The biological route for nitrogen gas entering the biosphere is reduction to ammonia by the nitrogenase enzyme, which is inactivated by oxygen. Three types of nitrogenase exist, the least-studied of which is the iron-only nitrogenase. The Anf3 protein in the bacterium *Rhodobacter capsulatus* is essential for diazotrophic (*i.e.* nitrogen-fixing) growth with the iron-only nitrogenase, but its enzymatic activity and function are unknown. Here, we biochemically and structurally characterize Anf3 from the model diazotrophic bacterium *Azotobacter vinelandii*. Determining the Anf3 crystal structure to atomic resolution, we observed that it is a dimeric flavocytochrome with an unusually close interaction between the heme and the FAD cofactors. Measuring the reduction potentials by spectroelectrochemical redox titration, we observed values of −420 ± 10 and −330 ± 10 mV for the two FAD potentials and −340 ± 1 mV for the heme. We further show that Anf3 accepts electrons from spinach ferredoxin and that Anf3 consumes oxygen without generating superoxide or hydrogen peroxide. We predict that Anf3 protects the iron-only nitrogenase from oxygen inactivation by functioning as an oxidase in respiratory protection, with flavodoxin or ferredoxin as the physiological electron donors.

## Introduction

The enzyme nitrogenase catalyzes biological nitrogen fixation and is found only in prokaryotes. Until the invention of the Haber–Bosch process, nitrogenase was by far the largest route for nitrogen to enter the biosphere. Modern agriculture depends on nitrogenous fertilizer produced by the Haber–Bosch process, but fertilizer use is polluting, and its production needs large amounts of methane and emits carbon dioxide. To minimize nitrogen pollution and to reduce the need for fertilizer, there is interest in expressing functional nitrogenase in crop plants, either directly or in prokaryotic symbiotes (1, 2). The iron-only alternative nitrogenase is a good candidate for expression (3), because it requires fewer genes than the more common MoFe nitrogenase. Nitrogenases are inactivated by oxygen, so understanding and overcoming this inhibition will be important for heterologous nitrogenase expression. The long-standing claim for the existence an oxygen-tolerant nitrogenase was recently debunked (4).

Nitrogenase has two components. The nitrogenase itself, where nitrogen is reduced to ammonia, is the MoFe protein, a heterotetramer of NifDK proteins. Nitrogen reduction occurs in MoFe at a complex metallocluster called FeMo-co that contains iron, sulfur, a molybdenum atom, and homocitrate. The second component is the dinitrogenase reductase or iron protein, which is a dimer of NifH, with a [4Fe-4S] cluster bridging the dimer and an ATPase site on each subunit. The iron protein is reduced by ferredoxin or flavodoxin (5) and passes electrons to the nitrogenase, hydrolyzing ATP to drive the reaction. The ideal reaction stoichiometry is given below, although under physiological conditions, more ATP is used and more hydrogen produced per nitrogen reduced (6).

$$\begin{array}{c} 8\;{\text{e}}^{-}+16\;\text{ATP}+{\text{N}}_{2}+8\;{\text{H}}^{+}\to 2\;{\text{NH}}_{3}+{\text{H}}_{2}+16\;\text{ADP}+16\;{\text{P}}_{\text{i}}\\ \text{Reaction}\;1 \end{array}$$

Two alternative nitrogenases are known, the vanadium or VFe nitrogenase and the iron-only, or FeFe nitrogenase, where the names reflect the metal replacing molybdenum in the FeMo-co. The alternative nitrogenases are encoded by *vnf* and *anf* genes, which are homologous to the MoFe enzyme *nif* genes. The VFe and FeFe nitrogenases have equivalent VFe-co and FeFe-co cofactors and their own specific iron proteins. The alternative nitrogenases are even more oxygen-sensitive than the MoFe enzyme (7, 8), have lower activity, consume more ATP, and produce proportionally more hydrogen. It is thought that the alternative nitrogenases are expressed when molybdenum or vanadium are limiting rather than for any other functional reason, and they are always found with the molybdenum nitrogenase.

Diazotrophs use several strategies to protect nitrogenase from oxygen (9). The two main mechanisms in *Azotobacter vinelandii* are respiratory protection and conformational protection. Conformational protection is mediated by the Shethna or FeSII protein. FeSII reversibly forms a ternary complex with the iron and MoFe proteins. The ternary complex is inactive but more resistant to oxygen than the individual nitrogenase subunits (10). The respiratory protection uses high respiratory rates to consume oxygen faster than it can inactivate nitrogenase. Respiratory protection mechanisms use alternative respiratory oxidases such as cytochrome *bd* and extra respiratory genes (11). So far, nothing is known of protective mechanisms specific to the alternative nitrogenases, and they have no known FeSII analogues.

The purple bacterium, *Rhodobacter capsulatus*, has an iron-only nitrogenase. Downstream of the *anfHDGK* structural genes are three cotranscribed ORFs called *anf1*, *anf2*, and *anf3* (Fig. S1). The *anf1* and *anf2* genes are homologous to *anfO* and *anfR* in *A. vinelandii* but are of unknown function. In *R. capsulatus* Anf3 is expressed in response to molybdenum starvation (12) and is required for nitrogen fixation by the iron-only nitrogenase (13, 14). The Anf3 in *Azotobacter* copurified with the iron-only nitrogenase of *A. vinelandii in vitro* and was characterized as a *b*-type cytochrome.^5^ Using the N-terminal sequence (Fig. S2), we identified the *b*-type cytochrome in the *A. vinelandii* genome (11) as an *anf3* (15).

Here, we have structurally and functionally characterized Anf3 from *A. vinelandii* and identified it as a flavocytochrome. The protein is a dimer and homologous to flavin-binding domains of the pyridoxamine 5′-phosphate oxidase family. Anf3 is a terminal oxygen oxidoreductase with ferredoxin or flavodoxin as the probable physiological electron donor. The terminal oxidase activity of the protein suggests an oxygen scavenging function to protect the iron-only nitrogenase. The closeness of the heme and FAD cofactors and biochemical and electrochemical data suggest that oxygen reduction to water is enabled by rapid electron transfer between the cofactors.

## Results

### Structure of Anf3

**Figure 1.**
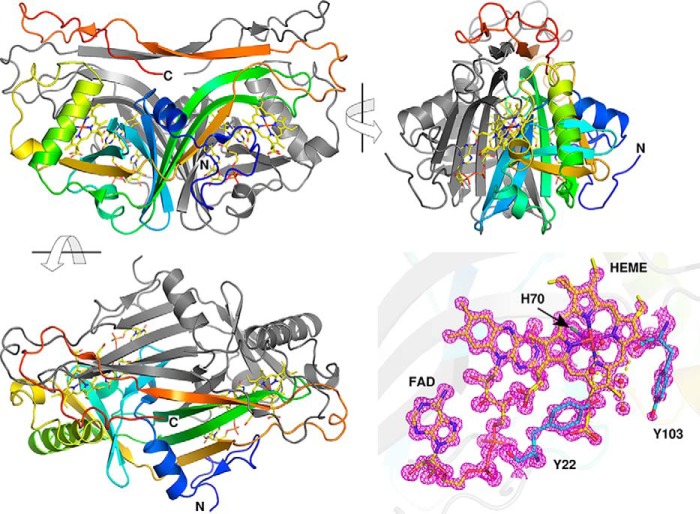
*A–C*, orthogonal views of the structure of Anf3, with the A chain ramped from *blue* at the N terminus to *red* at the C terminus and with the B chain in *gray. D*, electron density map for FAD and heme with the His^70^ and axial water, Tyr^22^, and Tyr^103^ with 2*F*_o_ − *F*_c_ density contoured at 3σ.

Anf3 was overexpressed in *Escherichia coli*, aerobically purified, and crystallized, and the structure determined to atomic resolution (Fig. 1 and Table 1). The asymmetric unit contained a dimer, the subunits are in similar conformations with a C_α RMSD_^6^ of 0.2 Å. Each monomer core is a split-barrel flavin-binding domain that binds an FAD and also a *b*-type heme. Two loops from residues 96–107 and 172 to the C terminus leave the core, interact with the other monomer, and loop back. A short two-strand symmetrical antiparallel β-sheet (residues 194–198 in both chains) links the two monomers. The dimer is tightly intertwined, and the cofactors interact with both monomers. Anf3 is most similar to the structure of MSMEG_4975 from *Mycobacterium smegmatis*, which also binds heme and FAD, with 35% identity and 2.6 Å RMSD. It was reported that the heme of MSMEG_4975 could be reversibly reduced by dithionite, but a function for the protein was not determined (16). Anf3 also superposes with the structure of the *E. coli* flavoenzyme pyridoxine 5′-phosphate oxidase (17) (Fig. S3) (C_α_ 2.1 Å RMSD over 111 residues). The Anf3 heme cofactor is in an equivalent position to the product pyridoxal 5′-phosphate in the pyridoxine 5′-phosphate oxidase structure.

**Table 1:** **Data collection and refinement statistics for Anf3** The statistics were calculated by PHENIX (51). Statistics for the highest-resolution shell are shown in parentheses. CC* = (CC_½_/(1 + (CC_½_)^0.5^)^0.5^. The data were integrated as far as 0.99 Å (the corner of the detector) but were only 20% complete at this resolution, giving 76% completeness overall. However, the resolution at the maximum inscribed circle was 1.16 Å with completeness 89%. Beamline geometry prevented the collection of a complete data set at 0.99 Å at the wavelength used. Individual anisotropic B-factors were refined.

	Anf3 (Protein Data Bank code 6RK0)
Wavelength (Å)	0.97625
Resolution range	32.24–0.99 (1.025–0.99)
Space group	P2_1_
Unit cell	48.97, 89.87, 59.03, 90, 109.06, 90
Total reflections	659,720 (10,253)
Unique reflections	203,126 (5207)
Multiplicity	3.2 (2.0)
Completeness (%)	76.02 (19.54)
Mean *I*/σ(*I*)	15.65 (1.10)
Wilson B-factor	11.45
*R*_merge_	0.03536 (0.7275)
*R*_meas_	0.04221 (0.9324)
*R*_pim_	0.02278 (0.5745)
CC_½_	0.999 (0.503)
CC*	1 (0.818)
Reflections used in refinement	203,119 (5202)
Reflections used for *R*_free_	10,214 (262)
*R*_work_	0.1236 (0.2901)
*R*_free_	0.1379 (0.2711)
CC_work_	0.975 (0.725)
CC_free_	0.975 (0.778)
Number of non-hydrogen atoms	4411
Macromolecules	3511
Ligands	288
Solvent	612
Protein residues	428
Root mean square bonds	0.013
Root mean square angles	1.33
Ramachandran favored (%)	98.82
Ramachandran allowed (%)	1.18
Ramachandran outliers (%)	0.00
Rotamer outliers (%)	0.00
Clashscore	4.98
Average B-factor	15.66
Macromolecules	14.05
Ligands	11.17
Solvent	27.01

The heme iron is ligated by a proximal histidine, His^70^, and an axial water molecule. Two conserved residues, Arg^21^ and Lys^170^ hydrogen-bond the heme propionates, stabilizing the heme–protein complex and controlling heme poise (18). The electron-withdrawing character of the hydrogen bonding contributes to the modulation of the heme electron density and influences the binding of ligands, such as oxygen, to the heme (19). The heme porphyrin ring is 3.4 Å from the isoalloxazine ring of the FAD at the closest point, consistent with a π-stacking interaction. Such a close interaction of the heme and flavin is unknown outside this class of flavocytochromes (20) and is relevant to our proposed mechanism of charge accumulation in the catalytic cycle. A network of hydrogen bonds links the heme axial water to the bulk solvent. Residues Tyr^103^, Asn^104^, Phe^105^, and Asn^106^ form a loop surrounding the heme axial water molecule. Through their side chains or via carbonyl groups, they create a network of hydrogen bonds (Fig. S4) that could stabilize intermediates in the catalytic cycle, provide routes for proton movement, and facilitate product water release from the active site. The active site cavity is filled with crystallographic waters and is open to the solvent. At the entrance to the active site there is a tyrosine (Tyr^22^) that is hydrogen-bonded to a water molecule that forms part of a chain of two water molecules hydrogen-bonded to the heme axial water. This network is a candidate for a proton relay, necessary for rapid delivery of protons to the substrate oxygen when it is reduced.

### Anf3 cofactor reduction potentials

**Figure 2.**
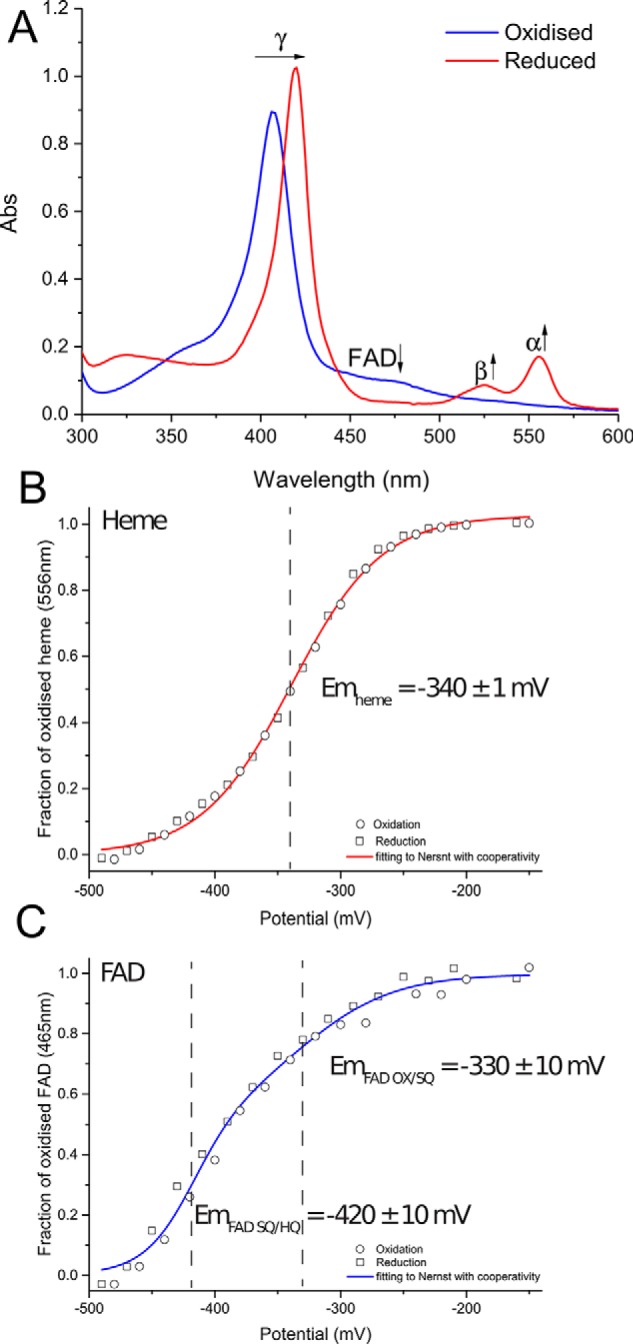
*A*, UV-visible spectra for as-prepared oxidized and reduced Anf3 from the titration end point. *B* and *C*, spectroelectrochemical redox titration of Anf3, following changes in absorbance at 556 nm showing the midpoint potential of the heme in Anf3 (*B*) and at 465 nm showing the midpoint potentials of the FAD in Anf3 (*C*). The final midpoint potential values shown are averages from the reductive and oxidative titrations of Anf3.

The UV-visible spectra of oxidized and reduced Anf3 (Fig. 2*A*) show features of both the *b*-type heme and the FAD. The absorption maximum at 407 nm, corresponding to the oxidized heme γ-band, shifted to 420 nm on reduction. The heme α and β-bands are in the reduced spectrum at 525 and 556 nm, respectively, but are not visible in the oxidized form. The presence of a small absorption peak at 625 nm suggests that the heme iron is in the high-spin state even though the crystal structure shows the heme iron coordinated by His^70^ and water. There is a shoulder at 465 nm in the oxidized spectrum corresponding to the FAD, which disappears on reduction. The lack of a resolved peak for the FAD is probably due to spectral interference from the heme and effects of the interaction between the heme and the FAD. The reduced spectrum with carbon monoxide bound (Fig. S5) shows that diatomic molecules can displace the water bound to the reduced heme and is consistent with a *b*-type cytochrome that can bind oxygen.

The reduction potential of the cofactors was determined by spectroelectrochemical redox titration. The fraction of oxidized heme (Fig. 2*B*) and oxidized FAD (Fig. 2*C*) were determined as a function of the potential, using their absorbances at 556 and 465 nm, respectively. The influence of the spectral overlap at these wavelengths was taken into account by also measuring other wavelengths where such interference was absent or minimized (Fig. S6 and “Experimental procedures”).

The midpoint potential of the heme in Anf3 was −340 ± 1 mV at pH 7.8 at 25 °C. The FAD had two midpoint potentials, corresponding to a two sequential one-electron reductions of the FAD (Fig. S6): one assigned to the FAD oxidized (FAD_ox_) to FAD semiquinone (FAD_sq_) transition at −330 ± 10 mV and one assigned to the FAD_sq_ to FAD hydroquinone (FAD_hq_) transition at −420 ± 10 mV at pH 7.8 at 25 °C. The experimental redox titration curves for the heme and FAD were distorted (Fig. 2 and Fig. S7) compared with a standard one-electron Nernst process (21, 22), which we modeled using a fractional apparent number of electrons transferred, as an indicator of cooperativity (11). We attribute this distortion to the proximity of the heme and the FAD, which leads to cooperative effects on the redox properties. The addition of the first electron will be favored by being shared across the two cofactors, whereas the addition of subsequent electrons will become more difficult because of the electrostatic repulsion between the cofactors. For example the addition of a third electron to fully reduce the FAD to the hydroquinone will be very difficult, resulting in the decrease of its reduction potential. This will in turn make the FAD_hq_ a strong reductant leading to fast electron transfer to the heme, necessary for the rapid accumulation of the four reducing equivalent needed for the reduction of oxygen to water (Fig. S7).

### Ferredoxin-dependent reduction of heme in Anf3

**Figure 3.**
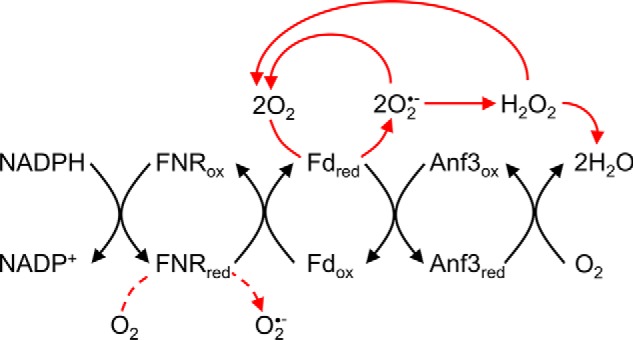
**Reaction scheme for ferredoxin-dependent NADPH reduction of Anf3 and cytochrome *c*.** Potential side reactions involving superoxide and hydrogen peroxide are shown in *red*.

To determine the catalytic activity of Anf3, we had to provide an appropriate source of low-potential electrons. We designed an assay system in which ferredoxin-NADP^+^ reductase (FNR) coupled NADPH oxidation to the reduction of ferredoxin, which was then used to reduce Anf3 (Fig. 3). Even in the absence of Anf3, background oxidation of NADPH was observed, because it could reduce ferredoxin, which in turn could donate electrons to oxygen, producing superoxide. The addition of Anf3 increased the rate of NADPH oxidation by 1.3 times, from 3.6 to 4.8 nmol NADPH/min, by competing with oxygen for the reduced ferredoxin (Fig. S8 and Table S1). Reduced Anf3 did not accumulate during aerobic NADPH oxidation, indicating that Anf3 donates electrons to another terminal acceptor. This biochemical observation, together with our structural and genetic analysis, suggested that oxygen is the terminal electron acceptor of the reaction catalyzed by Anf3.

**Figure 4.**
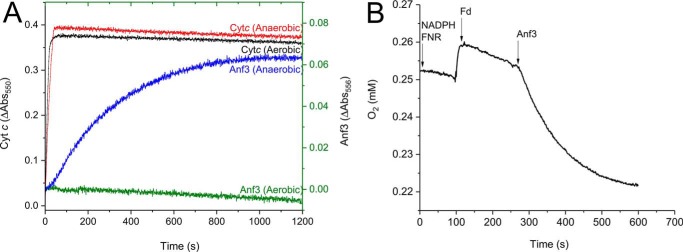
*A*, kinetics of heme reduction in Anf3 and cytochrome (*Cyt*) *c* in aerobic and anaerobic conditions. The kinetics of heme reduction were measured at 556 nm for Anf3 and 550 nm for cytochrome *c. Traces* are the means of three replicates. *B*, oxygen consumption activity of Anf3 in the coupled reaction system. The reaction was started with NADPH and FNR, which consume a small amount of oxygen. Fd was then added, giving more consumption of oxygen via the superoxide side reaction pathway. Anf3 was added last, triggering a faster rate of oxygen reduction. The mean of three replicates is shown.

To investigate this hypothesis, the kinetics of Anf3 heme reduction were measured aerobically and anaerobically. Cytochrome *c* was used as a control electron acceptor because its six-coordinated heme does not reduce oxygen (23). In the absence of oxygen, heme reduction was observed for Anf3 and cytochrome *c* (Fig. 4*A*). This showed that reduced ferredoxin can donate electrons to both proteins. The reduction of cyt *c* was much faster than Anf3, probably because the Δ*E* is more favorable for cyt *c* (∼670 mV) compared with Anf3 (∼150 mV). When the experiment was performed aerobically, we saw no Anf3 reduction, whereas cyt *c* was still rapidly reduced (Fig. 4*A*). This showed that Anf3 donated electrons to oxygen when it was present, and so Anf3 remained oxidized under these conditions. The reduction of Anf3 heme in the anaerobic assay was slow (Fig. 4*A*); the result of the small driving force as the potential of spinach ferredoxin (−401 mV) (24) is similar to the potential of the FAD bound to Anf3.

### Consumption of oxygen by Anf3

To confirm that oxygen is reduced by Anf3, oxygen consumption in the assay was measured with a Clark-type electrode (Fig. 4*C*). Background oxygen reduction was observed when either NADPH:FNR or NADPH:FNR:Fd were added to the buffer. Slow reduction of oxygen to superoxide by reduced ferredoxin is well-known. When Anf3 was added to the reaction, the rate of oxygen consumption increased 5-fold, from 2.4 nmol min^−1^ for NADPH:FNR:Fd to 14 nmol min^−1^ for NADPH:FNR:Fd:Anf3, confirming that reduced Anf3 can reduce oxygen. Reduced ferredoxin is produced slowly in the assay, so Anf3 reduction by ferredoxin is likely to be limiting rather than oxygen reduction; therefore the oxygen reduction rate is not a *V*_max_, and it was not possible to derive Michaelis–Menten parameters for oxygen reduction catalysis.

### Measurement of superoxide and hydrogen peroxide production by Anf3

**Figure 5.**
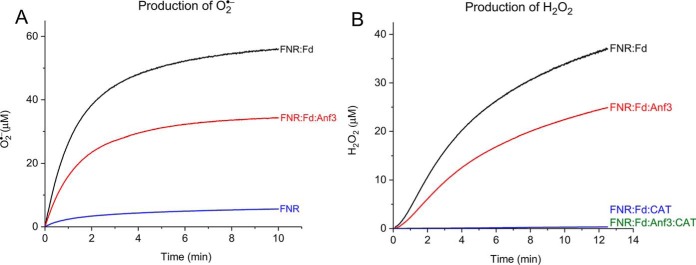
**Formation of superoxide and hydrogen peroxide in the coupled assay in the presence and absence of Anf3.** *A*, the rates of superoxide production measured using NBT at 560 nm. *B*, the rates of hydrogen peroxide production measured using Amplex red dye at 571 nm. The means of three replicates are shown.

Oxygen is reduced to water by four electrons, but incomplete reduction generates reactive oxygen species, which are harmful. Superoxide, hydrogen peroxide, and the hydroxyl radical are the products of one, two, and three electron reductions of oxygen, respectively. Superoxide was detected using nitroblue tetrazolium (NBT), and the kinetics showed that superoxide was generated by reduced ferredoxin donating electrons to oxygen. The addition of Anf3 led to a decrease in both the rate and amount of superoxide formation (Fig. 5*A* and Table S2). A similar trend was observed when measuring the rates of formation of hydrogen peroxide with Amplex Red dye (Fig. 5*B*). The hydrogen peroxide detected is probably the product of the spontaneous disproportionation of superoxide, and therefore its formation depends on the formation of superoxide. As observed for superoxide, the formation of hydrogen peroxide was detected in the presence of NADPH:FNR:Fd, and the addition of Anf3 decreased both the rate and the amount of hydrogen peroxide formed. The decrease in both superoxide and hydrogen peroxide production with Anf3 could be because the Anf3 competes with oxygen for the electrons from reduced ferredoxin, bypassing the NADPH:FNR:Fd:O_2_ superoxide generation pathway. Alternatively, one or more of superoxide and hydrogen peroxide could themselves be alternative electron donors to Anf3, with oxygen again the final product. These results showed that neither superoxide nor hydrogen peroxide are detectable products of oxygen reduction by Anf3 and that it is most plausible that a four-electron, four-proton reduction of oxygen to water occurs.

## Discussion

Anf3 belongs to a family of flavin-binding proteins, many of which do not bind heme. Unlike most members of this family, Anf3 is a flavocytochrome, binding a heme, where some other members of the family bind a substrate, as in pyridoxine 5′-phosphate oxidase (17). Anf3 is associated with the iron-only nitrogenase. The iron-only nitrogenase is inactivated by oxygen, so must be protected from it, particularly in the obligate aerobe *A. vinelandii*. There are relatively few sequenced genomes with the iron-only nitrogenase (25), of those known, only the Proteobacteria have Anf3 (Fig. S1). Organisms with iron-only nitrogenase but no Anf3 have other potential mechanisms to remove oxygen, such as flavo-diiron oxidase proteins. Two flavo-diiron proteins are specifically expressed in nitrogen-fixing heterocysts in the cyanobacterium *Anabaena* and are required for oxic diazotrophic growth (26).

Anf3-like genes are distributed widely in nondiazotrophic bacteria. These bacteria have other oxygen-sensitive enzymes, such as carbon monoxide dehydrogenase, hydrogenases, and formate dehydrogenases, which may also require Anf3-like activity to protect them from oxygen. Apart from oxygen removal, another possible function of Anf3 homologues could be to reduce NO, because the chemistry is similar to oxygen reduction. An NO detoxification function is proposed for other oxidases such as the flavo-diiron oxidases (27), and the function of *E. coli* flavohemoglobin is to reduce NO to nitrate (28).

The short distance between the heme and the FAD in Anf3 has only been seen in this structure and its homologues. Close proximity between redox cofactors has mechanistic importance in a class of tetraheme cytochromes including flavocytochrome *c*_3_ and fumarate reductase (21, 22). These are tetraheme proteins in which two of the heme molecules are only ∼3.9 Å apart, giving cooperative effects in electron transfer and leading to the rapid sequential donation of two electrons to the bound FAD. This decreases the probability of forming the FAD semiquinone and therefore prevents one-electron oxygen reduction and superoxide formation. Similar cooperative electron transfer effects were observed in the redox titrations of Anf3 (Fig. 3 and Fig. S7). We propose that the cooperativity is important in the catalytic mechanism of oxygen reduction, by allowing rapid and sequential electron transfer to the heme-bound oxygen. The heme reduction potential determined from the redox titrations is unusually low compared with other oxidases, where it ranges from −59 mV in *cbb*_3_ oxidase to +365 mV in bovine heart cytochrome *c* oxidase (29, 30) The high potentials in the terminal respiratory oxidases are required because oxygen reduction is linked to energy-conserving proton translocation (31). In contrast, Anf3 is an oxygen scavenger working in a low oxygen environment, where the reaction is accelerated by a higher driving force, and so low reduction potentials for the heme and the flavin are used. The rapidity of the reaction would prevent the release of reactive oxygen species intermediates and make the net reaction irreversible.

To determine whether water or superoxide or hydrogen peroxide was the product of oxygen reduction, we measured superoxide and hydrogen peroxide formation. We observed background formation of both superoxide and hydrogen peroxide, but upon the addition of Anf3, superoxide and hydrogen peroxide formation decreased, both in rates and total amounts. Anf3 was not acting as a superoxide dismutase, because this would increase hydrogen peroxide production, so we conclude that Anf3 was directly reducing oxygen to water.

The physiological electron donor to Anf3 is unknown, but *A. vinelandii* has several low-potential ferredoxin and flavodoxin isoforms that are plausible candidates (5). For example, ferredoxin-1 contains one 3Fe4S and one 4Fe4S center with reduction potentials of −425 and −647 mV, respectively (32), and both clusters of this protein could donate to Anf3. The electron donors to the nitrogenase are also potential donors to Anf3, which is consistent with Anf3 being active when nitrogenase is active.

The combination of the biochemical, electrochemical, and structural results show that Anf3 catalyzes the reduction of oxygen to water. We expect the catalytic mechanism to be similar to that of other heme containing oxidases (33, 34) because four electrons can be accumulated: two on the FAD and two on the heme, and then rapidly donated to the bound oxygen for its four-electron reduction to water. Sequential electron transfer from the physiological donor will lead to the formation of the fully reduced FAD and Fe^2+^ heme. Oxygen will bind with high affinity to the ferrous heme and upon protonation and release of a water molecule, the ferryl Fe(IV)=O^2−^, will be formed. Protonation will control the release of the second water molecule, whereas one electron donated by the physiological donor will lead to the formation of the resting state with FAD_ox_ and Fe^3+^. To decrease the probability of releasing any intermediate ROS species, four electrons must be provided rapidly and concertedly to the bound oxygen. These can be provided by the reduced FAD and heme, but we cannot exclude the contribution of either of two tyrosines, Tyr^22^ and Tyr^103^, close to the heme. However, they are not conserved (alignment in Fig. S3), so this cannot be the case for all homologues. Tyr^22^ is probably part of a proton relay through the hydrogen-bonded water chain in the active site cavity. Controlling protonation is crucial to the catalytic cycle, and the network of hydrogen bonds in the active site will be important in stabilizing the intermediates in the catalytic cycle and in facilitating the release of the water molecules generated as products. Hydrogen-bonding networks via water molecules are also observed around the heme propionates and the His^70^ acting as a fifth ligand. This suggests possible redox-tuning playing a role together with the control protonation. Because the FAD molecules in the two monomers are only 15 Å apart, we cannot exclude the possibility of intermonomers electron transfer because this would occur in the microsecond time scale (35). A probable physiological donor, the low potential ferredoxin-1 of *A. vinelandii* has two iron-sulfur clusters and so could donate two electrons rapidly, further reducing the chance of releasing reactive oxygen species intermediates. Nevertheless, the suggested mechanism would also work well with sequential one-electron reduction events from the physiological donor.

If Anf3 functions in a low oxygen environment, it must have a high oxygen affinity. Low potential heme proteins such as cytochromes P450 have submicromolar binding affinities for oxygen (36, 37). The Anf3 hemes are predicted to be low spin Fe^2+^ and so should have a high affinity for oxygen. In combination, the high driving force and high affinity for oxygen would make Anf3 an effective oxygen remover at low oxygen concentrations, where the oxygen concentration is already decreased by lower affinity respiratory oxidases. The most likely role of Anf3 is in protecting the iron-only nitrogenase from oxygen. Another possible function is to protect the cell from the nitrogenase. A lesser known function of the FeSII protein is to protect the cell from reactive oxygen species generated by oxygen and the nitrogenase proteins (38) It may be that Anf3 is required to prevent cascades of free radicals produced by oxygen reacting with the AnfHDK proteins.

The functional expression of nitrogenase in eukaryotes such as crop plants has many problems to be solved. One is the difficulty of assembling the complex cluster where nitrogen is reduced. The second is protecting the nitrogenase from oxygen in the aerobic plant. The alternative iron-only nitrogenase has a simpler assembly pathway than the molybdenum enzyme, requiring fewer genes, so is attractive as an engineering target (3). In this work, we describe the Anf3 protein that is essential for iron-only nitrogenase function in a purple bacterium and that has oxidase activity. Anf3 is therefore a promising candidate to enhance functional nitrogenase expression in heterologous systems.

## Experimental procedures

### Anf3 expression and structure solution

Recombinant Anf3 was overexpressed in *E. coli*. The His-tagged protein was purified by affinity and size-exclusion chromatographies and crystallized by hanging-drop vapor diffusion. The protein as purified contained heme and FAD. The structure was solved by X-ray crystallography (Protein Data Bank code 6RK0).

### Anf3 kinetics measurements

All kinetic measurements were carried out at 25 °C in 50 mm Tris-HCl (pH 7.8) using a UV-250 IPC spectrophotometer (Shimadzu) as described previously (39). The assay used a 10-mm-path length quartz cuvette, with a final volume of 100 μl, with 10 μm of ferredoxin (from *Spinacia oleracea*; Sigma), 0.01 units of FNR (from *S. oleracea*; Sigma), and 50 μm Anf3. The reaction was started by the addition of NADPH to 200 μm. NAPDH oxidation was followed at 340 nm. Anf3 heme reduction was followed at 556 nm. Background rates of ferredoxin reduction were subtracted to obtain the final rate of oxygen reduction by Anf3. The reduction of oxygen by Anf3 via the coupled assay was followed by a Clark-type oxygen electrode in a 1-ml thermostatted (25 °C) cuvette. Anaerobic assays used the pyranose oxidase catalase system to remove oxygen, with 7.5 units ml^−1^ of recombinant *Coriolus sp.* pyranose oxidase (Sigma), 1 kU ml^−1^ of catalase (Sigma), and 50 mm glucose (40). Superoxide formation by the coupled assay system was measured spectrophotometrically by measuring the reduction of nitrotetrazolium blue chloride (NBT) to blue formazan at 560 nm (41). Superoxide reacts with NBT with 2:1 stoichiometry to produce formazan (ϵ_560_ = 15 mm^−1^ cm^−1^) (42). NBT to 100 μm was added to the standard mixture, and the reaction was started by adding NADPH to 200 μm. The background reduction reaction in the presence of all components before the addition of ferredoxin and Anf3 was negligible. Hydrogen peroxide formation in the coupled assay system was measured with Amplex Red (100 μm) at 571 nm in the standard mixture and horseradish peroxidase (0.2 unit ml^−1^) (43). Amplex red reacts with hydrogen peroxide with 1:1 stoichiometry producing resorufin (ϵ_571_ = 58 mm^−1^ cm^−1^) (44). The reaction was started by the addition of NADPH to 200 μm. The background reduction reaction in the presence of catalase alone (1 kU ml^−1^) was negligible.

### Spectroelectrochemistry of Anf3

Spectroelectrochemical titrations were used to determine the midpoint potentials of the individual cofactors of Anf3. The titrations were performed in an optically transparent thin-layer electrochemical cell by monitoring redox-induced changes in absorbance at wavelengths corresponding to characteristic spectroscopy features of the cofactors. The optically transparent thin-layer electrochemical cell (path length, 0.5 mm) had a lid through which a gold mesh working electrode, a Pt counter electrode, an Ag/AgCl reference electrode, and an argon purging needle were inserted. The reaction mixture was prepared in 50 mm Tris-HCl (pH 7.8) to a final volume of 500 μl with Anf3 to 250 μm. The following redox mediators were used in all titrations: 5 μm methyl viologen (*E*_m_ = −446 mV), 5 μm benzyl viologen (*E*_m_ = −358 mV), and 10 μm anthraquinone-2-sulfonate (*E*_m7.5_ = −225 mV) covering the potential range between −225 and −446 mV. Within the cell, the mixture was deoxygenated by the pyranose oxidase catalase system, and a flow of argon (2 min of flow at 15-min intervals) maintained the anaerobic condition. The absorbance contributions of the mediators during the titration were subtracted by performing a blank titration without protein. The absorbance values as a function of the applied potential, at the chosen wavelengths, where subtracted from the values measured in the presence of the protein.

For the heme, the Soret β-band at 556 nm was used, and it showed a monotonic increase in intensity upon reduction. For the FAD, the absorbance at 465 nm was used, and it showed a monotonic decrease upon reduction from quinone to semiquinone and then to hydroquinone. To assess the potential spectroscopic interference of the cofactors on each other, alternative wavelength were also monitored. Absorbance at 420 nm minus the absorbance at 407 nm was monitored because the heme Soret γ-band shows a strong band shift upon reduction and because the heme extinction coefficient is 10 times higher than the FAD in this region. The identification of unique absorption feature of the FAD requires knowledge of the nature of the semiquinone formed during reduction, because the red anion form has different spectroscopic characteristics when compared with the blue neutral form. We observed spectroscopic changes during the titration at 600 nm, where no contribution from the heme Soret is expected, that are consistent with the formation of the neutral blue semiquinone. Furthermore, to obtain the FAD neutral semiquinone, protonation of the N5 atom must occur. In other flavoproteins, the proton on N5 is stabilized by hydrogen bonding through a protein residue. The backbone nitrogen of Phe^95^ in Anf3 can hydrogen bond the FAD N5; this, together with characteristic spectroscopic changes at 600 nm, suggested that the neutral semiquinone was formed on reduction.

The titrations were carried out in both the reducing and oxidizing directions to show the thermodynamic reversibility. *E*_m_ values were determined by fitting rearranged Nernst equations adapted from Turner *et al.* (54) with OriginPro (OriginLab, Northampton, MA). For the heme, Equation 1 was used for one transition from Fe^3+^ to Fe^2+^. For the FAD, Equation 2 was used for two consecutive redox transitions corresponding to the FAD transitions from oxidized to semiquinone and from semiquinone to hydroquinone. In the equations *f*_ox_ represents the fraction of oxidized species, *a* is the fraction of the quinone to semiquinone transition, and *b* is the fraction of the semiquinone to hydroquinone transition. *E*_h_, *E*_Q-SQ_, and *E*_Q-HQ_ are the midpoint potentials for the heme, the quinone–semiquinone, and semiquinone–hydroquinone redox couples respectively; *n*_a_, *n*_b_, and *n*_c_ are the apparent number of electrons transferred and give an indication of the degree of cooperativity; *R* is the gas constant; *T* is the absolute temperature; and *F* is the Faraday constant. For the fitting of the data without cooperativity presented in Fig. S6, Equation 1 was used with fixed *n*_c_ = 1. The midpoint potential values and the standard errors are given from averages from the oxidative and reductive titrations. All potentials are relative to the standard hydrogen electrode.

(Eq. 1) $$f_{\text{ox}}=\frac{1}{1+\exp\left[\frac{\left(E-E_{\text{h}}\right)}{\frac{\text{RT}}{n_{\text{c}}F}}\right]}$$

(Eq. 2) $$f_{\text{ox}}=\frac{a}{1+\exp\left[\frac{\left(E-E_{\text{Q}-\text{SQ}}\right)}{\frac{\text{RT}}{n_{\text{a}}F}}\right]}+\frac{b}{1+\exp\left[\frac{\left(E-E_{\text{SQ}-\text{HQ}}\right)}{\frac{\text{RT}}{n_{\text{b}}F}}\right]}$$

### Anf3 cloning

The unpublished N-terminal sequence produced in 1993 from Anf3 (then called cytochrome *b′*) purified from *A. vinelandii* RP306 (45) (TNRTDEEYPEAPSDRTCVKSYAS) was an 86% identical match in the *Azotobacter* genome to Avin_49040 (Fig. S2). The *anf3* gene (Avin_49040) was amplified from *A. vinelandii* (DSM 2289) genomic DNA, with primers (Anf3 forward, CATCATCATG GTCTGGTTCC GCGTGGATCC ATGACGAACC GGACGGACGAC; and Anf3 reverse, TCCATGGTAC CAGCTGCAGA TCTCGAGCTC TCAGCCCAAG CGGAATTTCA ATATGTC) with the necessary overhangs for Gibson assembly (46) into a modified pRSET-A expression vector (47). The modified pRSET-A backbone was amplified with (prset forward, GAGCTCGAGAT CTGCAGCTG; and prset reverse, CGCGGAACCAG ACCATGATG), and the insert and backbone combined with Gibson assembly to give the kanamycin-resistant pRSET-A-anf3 vector, encoding a protein with a His_6_ tag at the N terminus followed by a thrombin cleavage site then *anf3*. The sequence was checked by Sanger sequencing.

### Anf3 purification and crystallization

The pRSET-anf3 plasmid was transformed into chemically competent *E. coli* KRX cells (Promega). For expression, the cells were cultured in 1.0 liter of Terrific broth at 37 °C to an *A*_600_ of 0.8. Protein expression was induced with 0.1% (w/v) rhamnose (Sigma), and the cells were grown at 18 °C for an additional 15 h. The cells were harvested by centrifugation at 4,500 × *g*, resuspended in 100 mm Tris-HCl (pH 7.9) and 150 mm NaCl (buffer A) at 1 g ml^−1^, and lysed by sonication for 10 min (2-s sonication with 2-s pause) at 55% amplitude. Clear cell lysate was collected after ultracentrifugation (142,000 × *g*, 30 min) and applied onto two spin columns containing 3 ml of super nickel–nitrilotriacetic acid–agarose resin (Generon) each, pre-equilibrated with buffer A. Imidazole was avoided in the purification, because it binds tightly to the *b*-type heme. Anf3 was eluted with direct digestion of the resin-bound His-tagged protein by 1 mg of thrombin from bovine plasma (Sigma) in 15 ml of buffer A at 4 °C overnight. The eluted protein was concentrated to 1 ml and loaded onto a Superdex 200 HiLoad column (GE Healthcare) pre-equilibrated with buffer A. Purified Anf3 fractions were pooled, concentrated, and stored at 4 °C. Typically, a 1-liter culture yielded 60 mg of Anf3. The protein as isolated contained heme and FAD. Vapor diffusion crystallization experiments were performed in a 24-well hanging-drop plate. A volume of 1 μl of protein was mixed with 1 or 2 μl of precipitant solution (25.5% PEG 4000, 170 mm ammonium sulfate) and suspended over 200 μl of precipitant solution and incubated at 16 °C. Crystals appeared after a few days and grew to full size over 2 weeks.

### Anf3 structure solution

Crystals of Anf3 were cryoprotected in the mother liquor with 30% (v/v) glycerol added, and then flash-cooled in liquid nitrogen. X-ray diffraction data were collected on Beamline I03 at the Diamond Light Source. The data were processed in space group P2_1_ to 1.0 Å resolution with xia2 (48) and XDS (49) for integration. The structure was solved by molecular replacement in PHASER (50) with a model based on MSMEG_4975 (Protein Data Bank code 4YBR) (16). The structure was refined in Phenix (51) to a final *R*/*R*_free_ of 12.4/13.8% with cycles of model building in COOT (52). The heme groups were refined in two orientations with ∼180° rotation around the α,γ-meso axis (53). The minor orientation refined to 22% (A) and 18% (B) occupancy in the two chains. There was no observed effect of the heme orientation in the redox titrations. The data and refinement statistics are given in Table S1. The structure was deposited in the Protein Data Bank (code 6RK0).

## Author contributions

F. V. and J. W. M. formal analysis; F. V., B. V. K., C. A. R. C., A. F., and J. W. M. investigation; F. V., A. F., and J. W. M. writing-original draft; F. V., J. S., A. W. R., A. F., and J. W. M. writing-review and editing; J. S., A. W. R., A. F., and J. W. M. conceptualization; A. W. R. and J. W. M. funding acquisition; A. W. R., A. F., and J. W. M. project administration; A. F. validation; A. F. visualization; A. F. and J. W. M. methodology.

## Supplementary Material

Supporting Information

## Footnotes

5 J. Schumacher (1993) A novel cytochrome *b'* of *A. vinelandii* and its role in nitrogen fixation. Diplomarbeit, University of Cologne.

## Footnotes

6 The abbreviations used are:

RMSD: root-mean-square deviation
NBT: nitrotetrazolium blue chloride
FNR: ferredoxin-NADP^+^ reductase
Fd: ferredoxin.

## References

[1] Oldroyd G. E., and Dixon R. (2014) Biotechnological solutions to the nitrogen problem. Curr. Opin. Biotechnol. 26, 19–24 10.1016/j.copbio.2013.08.006 24679253

[2] López-Torrejón G., Jiménez-Vicente E., Buesa J. M., Hernandez J. A., Verma H. K., and Rubio L. M. (2016) Expression of a functional oxygen-labile nitrogenase component in the mitochondrial matrix of aerobically grown yeast. Nat. Commun. 7, 11426 10.1038/ncomms11426 27126134 PMC4855529

[3] Yang J., Xie X., Wang X., Dixon R., and Wang Y.-P. (2014) Reconstruction and minimal gene requirements for the alternative iron-only nitrogenase in *Escherichia coli*. Proc. Natl. Acad. Sci. U.S.A. 111, E3718–E3725 10.1073/pnas.1411185111 25139995 PMC4156695

[4] MacKellar D., Lieber L., Norman J. S., Bolger A., Tobin C., Murray J. W., Oksaksin M., Chang R. L., Ford T. J., Nguyen P. Q., Woodward J., Permingeat H. R., Joshi N. S., Silver P. A., Usadel B., et al (2016) *Streptomyces thermoautotrophicus* does not fix nitrogen. Sci. Rep. 6, 20086 10.1038/srep20086 26833023 PMC4735515

[5] Segal H. M., Spatzal T., Hill M. G., Udit A. K., and Rees D. C. (2017) Electrochemical and structural characterization of *Azotobacter vinelandii* flavodoxin II. Protein Sci. 26, 1984–1993 10.1002/pro.3236 28710816 PMC5606536

[6] Burgess B. K., and Lowe D. J. (1996) Mechanism of molybdenum nitrogenase. Chem. Rev. 96, 2983–3012 10.1021/cr950055x 11848849

[7] Moshiri F., Kim J. W., Fu C., and Maier R. J. (1994) The FeSII protein of *Azotobacter vinelandii* is not essential for aerobic nitrogen fixation, but confers significant protection to oxygen-mediated inactivation of nitrogenase *in vitro* and *in vivo*. Mol. Microbiol. 14, 101–114 10.1111/j.1365-2958.1994.tb01270.x 7830548

[8] Chisnell J. R., Premakumar R., and Bishop P. E. (1988) Purification of a second alternative nitrogenase from a nifHDK deletion strain of *Azotobacter vinelandii*. J. Bacteriol. 170, 27–33 10.1128/jb.170.1.27-33.1988 3121587 PMC210601

[9] Gallon J. R. (1992) Reconciling the incompatible: N_2_ fixation and O_2_. New Phytol. 122, 571–609

[10] Robson R. L. (1979) Characterization of an oxygen-stable nitrogenase complex isolated from *Azotobacter chroococcum*. Biochem. J. 181, 569–575 10.1042/bj1810569 518541 PMC1161196

[11] Setubal J. C., dos Santos P., Goldman B. S., Ertesvåg H., Espin G., Rubio L. M., Valla S., Almeida N. F., Balasubramanian D., Cromes L., Curatti L., Du Z., Godsy E., Goodner B., Hellner-Burris K., et al (2009) Genome sequence of *Azotobacter vinelandii*, an obligate aerobe specialized to support diverse anaerobic metabolic processes. J. Bacteriol. 191, 4534–4545 10.1128/JB.00504-09 19429624 PMC2704721

[12] Hoffmann M.-C., Wagner E., Langklotz S., Pfänder Y., Hött S., Bandow J. E., and Masepohl B. (2015) Proteome profiling of the *Rhodobacter capsulatus* molybdenum response reveals a role of IscN in nitrogen fixation by Fe-nitrogenase. J. Bacteriol. 198, 633–643 26644433 10.1128/JB.00750-15 PMC4751811

[13] Klipp W., Angermuller S., Astroth S., Kamp P.-B., Kern M., Kutsche M., Leimkuhler S., and Paschen A. (1995) Regulation of molybdenum and alternative nitrogenases in the photosynthetic purple bacterium *Rhodobacter capsulatus*. In Nitrogen Fixation: Fundamentals and Applications (Tikhonovich I. A., Provorov N. A., Romanov V. I., and Newton W. E., eds) pp. 201–206, Kluwer Academic Publishers, Dordrecht, The Netherlands

[14] Masepohl B., and Klipp W. (1996) Organization and regulation of genes encoding the molybdenum nitrogenase and the alternative nitrogenase in *Rhodobacter capsulatus*. Arch. Microbiol. 165, 80–90 10.1007/s002030050301

[15] Sicking C., Brusch M., Lindackers A., Riedel K.-U., Schubert B., Isakovic N., Krall C., Klipp W., Drepper T., Schneider K., and Masepohl B. (2005) Identification of two new genes involved in diazotrophic growth via the alternative Fe-only nitrogenase in the phototrophic purple bacterium *Rhodobacter capsulatus*. J. Bacteriol. 187, 92–98 10.1128/JB.187.1.92-98.2005 15601692 PMC538833

[16] Ahmed H., Carr P. D., Lee B. M., Afriat-Jurnou L., Mohamed A. E., Hong N.-S., Flanagan J., Taylor M. C., Greening C., and Jackson C. J. (2015) Sequence-structure-function classification of a catalytically diverse oxidoreductase superfamily in *Mycobacteria*. J. Mol. Biol. 427, 3554–3571 10.1016/j.jmb.2015.09.021 26434506

[17] Safo M. K., Musayev F. N., di Salvo M. L., and Schirch V. (2001) X-ray structure of *Escherichia coli* pyridoxine 5′-phosphate oxidase complexed with pyridoxal 5′-phosphate at 2.0 Å resolution. J. Mol. Biol. 310, 817–826 10.1006/jmbi.2001.4734 11453690

[18] Hunter C. L., Lloyd E., Eltis L. D., Rafferty S. P., Lee H., Smith M., and Mauk A. G. (1997) Role of the heme propionates in the interaction of heme with apomyoglobin and apocytochrome *b*_5_. Biochemistry 36, 1010–1017 10.1021/bi961385u 9033390

[19] Yoshimura H., Yoshioka S., Kobayashi K., Ohta T., Uchida T., Kubo M., Kitagawa T., and Aono S. (2006) Specific hydrogen-bonding networks responsible for selective O_2_ sensing of the oxygen sensor protein HemAT from *Bacillus subtilis*. Biochemistry 45, 8301–8307 10.1021/bi060315c 16819829

[20] Mowat C. G., Gazur B., Campbell L. P., and Chapman S. K. (2010) Flavin-containing heme enzymes. Arch. Biochem. Biophys. 493, 37–52 10.1016/j.abb.2009.10.005 19850002

[21] Coletta M., Catarino T., LeGall J., and Xavier A. V. (1991) A thermodynamic model for the cooperative functional properties of the tetraheme cytochrome *c*_3_ from *Desulfovibrio gigas*. Eur. J. Biochem. 202, 1101–1106 10.1111/j.1432-1033.1991.tb16476.x 1662600

[22] Pessanha M., Rothery E. L., Miles C. S., Reid G. A., Chapman S. K., Louro R. O., Turner D. L., Salgueiro C. A., and Xavier A. V. (2009) Tuning of functional heme reduction potentials in *Shewanella* fumarate reductases. Biochim. Biophys. Acta 1787, 113–120 10.1016/j.bbabio.2008.11.007 19081388

[23] Schejter A., Ryan M. D., Blizzard E. R., Zhang C., Margoliash E., and Feinberg B. A. (2006) The redox couple of the cytochrome *c* cyanide complex: the contribution of heme iron ligation to the structural stability, chemical reactivity, and physiological behavior of horse cytochrome *c*. Protein Sci. 15, 234–241 10.1110/ps.051825906 16434742 PMC2242453

[24] Aliverti A., Hagen W. R., and Zanetti G. (1995) Direct electrochemistry and EPR spectroscopy of spinach ferredoxin mutants with modified electron transfer properties. FEBS Lett. 368, 220–224 10.1016/0014-5793(95)00648-S 7628609

[25] McRose D. L., Zhang X., Kraepiel A. M., and Morel F. M. (2017) Diversity and activity of alternative nitrogenases in sequenced genomes and coastal environments. Front. Microbiol. 8, 267 28293220 10.3389/fmicb.2017.00267 PMC5328986

[26] Ermakova M., Battchikova N., Richaud P., Leino H., Kosourov S., Isojärvi J., Peltier G., Flores E., Cournac L., Allahverdiyeva Y., and Aro E.-M. (2014) Heterocyst-specific flavodiiron protein Flv3B enables oxic diazotrophic growth of the filamentous cyanobacterium *Anabaena* sp. PCC 7120. Proc. Natl. Acad. Sci. U.S.A. 111, 11205–11210 10.1073/pnas.1407327111 25002499 PMC4121841

[27] Kurtz D. (2007) Flavo-diiron enzymes: nitric oxide or dioxygen reductases? Dalt. Trans. 37, 4115–4121

[28] Gardner P. R., Gardner A. M., Martin L. A., and Salzman A. L. (1998) Nitric oxide dioxygenase: an enzymic function for flavohemoglobin. Proc. Natl. Acad. Sci. U.S.A. 95, 10378–10383 10.1073/pnas.95.18.10378 9724711 PMC27902

[29] Rauhamäki V., Bloch D. A., Verkhovsky M. I., and Wikström M. (2009) Active site of cytochrome *cbb*_3_. J. Biol. Chem. 284, 11301–11308 10.1074/jbc.M808839200 19252222 PMC2670135

[30] Melin F., Xie H., Meyer T., Ahn Y. O., Gennis R. B., Michel H., and Hellwig P. (2016) The unusual redox properties of C-type oxidases. Biochim. Biophys. Acta 1857, 1892–1899 10.1016/j.bbabio.2016.09.009 27664317

[31] Sharma V., Enkavi G., Vattulainen I., Róg T., and Wikström M. (2015) Proton-coupled electron transfer and the role of water molecules in proton pumping by cytochrome *c* oxidase. Proc. Natl. Acad. Sci. U.S.A. 112, 2040–2045 10.1073/pnas.1409543112 25646428 PMC4343153

[32] Iismaa S. E., Vázquez A. E., Jensen G. M., Stephens P. J., Butt J. N., Armstrong F. A., and Burgess B. K. (1991) Site-directed mutagenesis of *Azotobacter vinelandii* ferredoxin I: changes in [4Fe-4S] cluster reduction potential and reactivity. J. Biol. Chem. 266, 21563–21571 1657971

[33] Bloch D., Belevich I., Jasaitis A., Ribacka C., Puustinen A., Verkhovsky M. I., and Wikström M. (2004) The catalytic cycle of cytochrome *c* oxidase is not the sum of its two halves. Proc. Natl. Acad. Sci. U.S.A. 101, 529–533 10.1073/pnas.0306036101 14699047 PMC327181

[34] Rich P. (2017) Mitochondrial cytochrome *c* oxidase: catalysis, coupling and controversies. Biochem. Soc. Trans. 45, 813–829 10.1042/BST20160139 28620043

[35] Page C. C., Moser C. C., Chen X., and Dutton P. L. (1999) Natural engineering principles of electron tunnelling in biological oxidation–reduction. Nature 402, 47–52 10.1038/46972 10573417

[36] Addison A. W., and Burman S. (1985) Ligand-dependent redox chemistry of *Glycera dibranchiata* hemoglobin. Biochim. Biophys. Acta 828, 362–368 10.1016/0167-4838(85)90317-6

[37] Lewis D. F., and Sheridan G. (2001) Cytochromes P450, oxygen, and evolution. Sci. World J. 1, 151–167 10.1100/tsw.2001.22 12805700 PMC6084128

[38] Maier R. J., and Moshiri F. (2000) Role of the *Azotobacter vinelandii* nitrogenase-protective shethna protein in preventing oxygen-mediated cell death. J. Bacteriol. 182, 3854–3857 10.1128/JB.182.13.3854-3857.2000 10851006 PMC94562

[39] Zanetti G., and Curti B. (1980) [22] Ferredoxin-NADP^+^ oxidoreductase. Methods Enzymol. 69, 250–255 10.1016/S0076-6879(80)69024-7

[40] Swoboda M., Henig J., Cheng H.-M., Brugger D., Haltrich D., Plumeré N., and Schlierf M. (2012) Enzymatic oxygen scavenging for photostability without pH drop in single-molecule experiments. ACS Nano. 6, 6364–6369 10.1021/nn301895c 22703450 PMC3403312

[41] Nishikimi M., Appaji N., and Yagi K. (1972) The occurrence of superoxide anion in the reaction of reduced phenazine methosulfate and molecular oxygen. Biochem. Biophys. Res. Commun. 46, 849–854 10.1016/S0006-291X(72)80218-3 4400444

[42] Case C. L., Rodriguez J. R., and Mukhopadhyay B. (2009) Characterization of an NADH oxidase of the flavin-dependent disulfide reductase family from *Methanocaldococcus jannaschii*. Microbiology 155, 69–79 10.1099/mic.0.024265-0 19118348

[43] Kareyeva A. V., Grivennikova V. G., Cecchini G., and Vinogradov A. D. (2011) Molecular identification of the enzyme responsible for the mitochondrial NADH-supported ammonium-dependent hydrogen peroxide production. FEBS Lett. 585, 385–389 10.1016/j.febslet.2010.12.019 21168410 PMC3022077

[44] Al-Attar S., Yu Y., Pinkse M., Hoeser J., Friedrich T., Bald D., and de Vries S. (2016) Cytochrome *bd* displays significant quinol peroxidase. Sci. Rep. 6, 27631 10.1038/srep27631 27279363 PMC4899803

[45] Pau R. N., Eldridge M. E., Lowe D. J., Mitchenall L. A., and Eady R. R. (1993) Molybdenum-independent nitrogenases of *Azotobacter vinelandii*: a functional species of alternative nitrogenase-3 isolated from a molybdenum-tolerant strain contains an iron-molybdenum cofactor. Biochem. J. 293, 101–107 10.1042/bj2930101 8392330 PMC1134325

[46] Gibson D. (2011) Enzymatic assembly of overlapping DNA fragments. Methods Enzymol. 498, 349–361 10.1016/B978-0-12-385120-8.00015-2 21601685 PMC7149801

[47] Michoux F., Takasaka K., Boehm M., Nixon P. J., Murray J. W. (2010) Structure of CyanoP at 2.8 A: implications for the evolution and function of the PsbP subunit of photosystem II. Biochemistry 49, 7411–7413 10.1021/bi1011145 20698571

[48] Winter G. (2010) xia2: An expert system for macromolecular crystallography data reduction. J. Appl. Crystallogr. 43, 186–190 10.1107/S0021889809045701

[49] Kabsch W. (2010) XDS. XDS. Acta Crystallogr. D Biol. Crystallogr. 66, 125–132 10.1107/S0907444909047337 20124692 PMC2815665

[50] McCoy A. J., Grosse-Kunstleve R. W., Adams P. D., Winn M. D., Storoni L. C., and Read R. J. (2007) Phaser crystallographic software. J. Appl. Crystallogr. 40, 658–674 10.1107/S0021889807021206 19461840 PMC2483472

[51] Adams P. D., Afonine P. V., Bunkóczi G., Chen V. B., Davis I. W., Echols N., Headd J. J., Hung L. W., Kapral G. J., Grosse-Kunstleve R. W., McCoy A. J., Moriarty N. W., Oeffner R., Read R. J., Richardson D. C., et al (2010) *PHENIX*: a comprehensive Python-based system for macromolecular structure solution. Acta Crystallogr. D 66, 213–221 10.1107/S0907444909052925 20124702 PMC2815670

[52] Emsley P., Lohkamp B., Scott W. G., and Cowtan K. (2010) Features and development of COOT. Acta Crystallogr. D. Biol. Crystallogr. 66, 486–501 10.1107/S0907444910007493 20383002 PMC2852313

[53] Yamamoto Y., and La Mar G. N. (1986) Proton NMR study of dynamics and thermodynamics of heme rotational disorder in native and reconstituted hemoglobin A. Biochemistry 25, 5288–5297 10.1021/bi00366a045 3768348

[54] Turner K. L., Doherty M. K., Heering H. A., Armstrong F. A., Reid G. A., and Chapman S. K. (1999) Redox properties of flavocytochrome *c*_3_ from *Shewanella frigidimarina* NCIMB400. Biochemistry 38, 3302–3309 10.1021/bi9826308 10079073

