# Supplementary material for: A low-potential terminal oxidase associated with the iron-only nitrogenase from the nitrogen-fixing bacterium Azotobacter vinelandii
Source: J Biol Chem. 2019 May 1;294(24):9367–76. doi: 10.1074/jbc.RA118.007285 (PMC6579470; doi:10.1074/jbc.RA118.007285)
Supplement: Supporting Information [file supp_294_24_9367__index.html]

A low-potential terminal oxidase associated with the iron-only nitrogenase from the nitrogen-fixing bacterium Azotobacter vinelandii — Structure and function of Anf3 — A low-potential terminal oxidase associated with the iron-only nitrogenase from the nitrogen-fixing bacterium Azotobacter vinelandii — EDITORS' PICK: Structure and function of Anf3 — Supporting Information 

# A low-potential terminal oxidase associated with the iron-only nitrogenase from the nitrogen-fixing bacterium *Azotobacter vinelandii*

## Supporting Information

- Supporting Information (to be published online) - supplementary figures
