# Supplementary material for: A low-potential terminal oxidase associated with the iron-only nitrogenase from the nitrogen-fixing bacterium Azotobacter vinelandii
Source: J Biol Chem. 2019 May 1;294(24):9367–76. doi: 10.1074/jbc.RA118.007285 (PMC6579470; doi:10.1074/jbc.RA118.007285)
Supplement: Supporting Information [file supp_RA118.007285_142624_1_supp_320869_pqkxlv.docx]

Supplementary Information

**A low-potential terminal oxidase associated with the iron-only nitrogenase from the nitrogen-fixing bacterium *Azotobacter vinelandii***

Febin Varghese^a^, Burak Veli Kabasakal^ab^, Charles AR Cotton^ac^, Jörg Schumacher^a^, A William Rutherford^a^, Andrea Fantuzzi^a**^, James W Murray^a**^

a Department of Life Sciences, Imperial College, London, London SW7 2AZ, United Kingdom

b Current address: School of Biochemistry, University of Bristol, Biomedical Sciences Building, University Walk, Bristol, BS8 1TD, United Kingdom

c Current address: Max Planck Institute of Molecular Plant Physiology, Am Mühlenberg 1, 14476 Potsdam-Golm, Germany

** to whom correspondence should be addressed. Email: j.w.murray@imperial.ac.uk and a.fantuzzi@imperial.ac.uk

List of contents:

Supplementary tables: Table S1-Table S2

Supplementary figures: S1-S8

Supplementary References

| **Measurement** | **FNR:Fd**  nmol NADPH/min | **FNR:Fd:Anf3**  nmol NADPH/min | **Apparent specific activities**  nmol NADPH/min/mg Anf3 |
| --- | --- | --- | --- |
| **NADPH oxidation** | 3.6 ± 0.5 | 4.8 ± 0.3 | 4.8 ± 0.4 |
| **Oxygen reduction** | 2.4 ± 0.4 | 14.0 ± 2.0 | 9.2 ± 1.6 |

**Table S1. The rates of NADPH oxidation and oxygen reduction by reduced ferredoxin and Anf3**

The reduction of oxygen by Anf3 (FNR:Fd:Anf3) was measured by NADPH oxidation and Clark-type electrode using the coupled assay. The apparent specific activities are measured by the subtraction of FNR:Fd rates from FNR:Fd:Anf3 rates.

| **Measurement** | **FNR:Fd** | **FNR:Fd:Anf3** |
| --- | --- | --- |
| **NBT reduction**nmol $O_{2}^{\cdot-}$/min | 2.8 ± 0.2 | 1.7 ± 0.1 |
| **AXR reduction**  nmol H_2_O_2_/min | 0.57 ± 0.03 | 0.32 ± 0.04 |

**Table S2. The rates of ROS production by reduced ferredoxin (Fd) and Anf3**. The production of ROS by Anf3 was measured by NBT reduction for superoxide formation and AXR reduction for hydrogen peroxide formation. The background rate of ROS production by reduced ferredoxin was measured similarly for both assays.


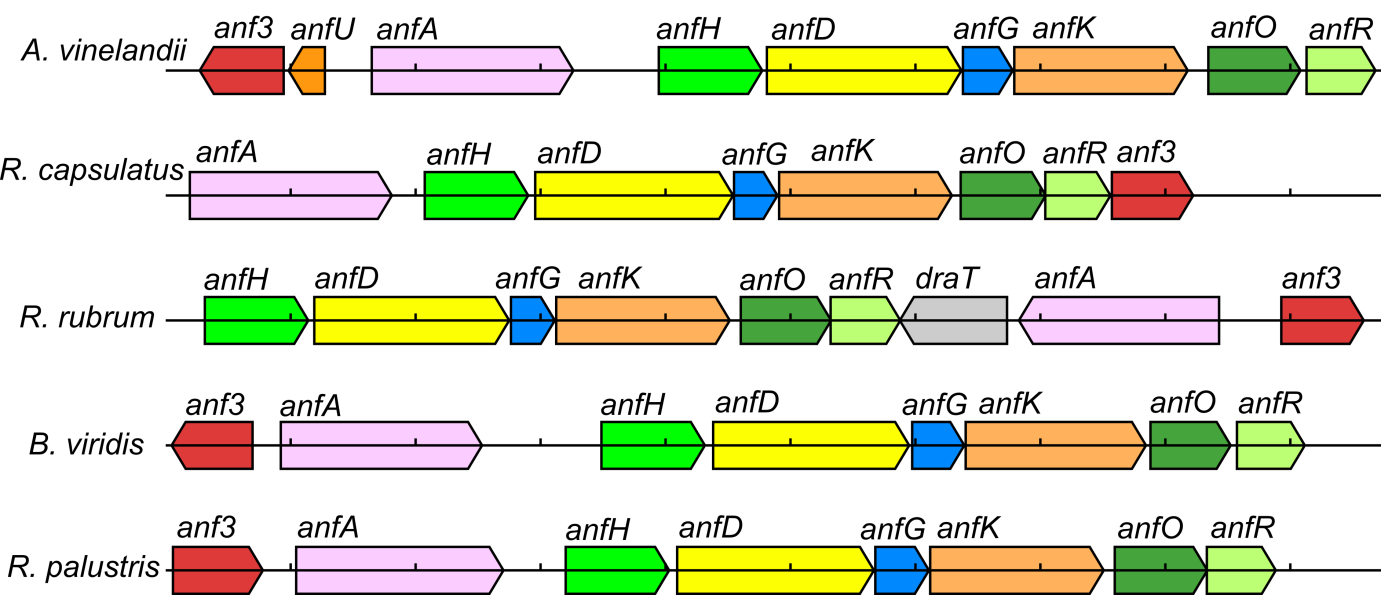


**Figure S1. Diagram of the *anf* operon** in the genomes of *Azotobacter vinelandii* (1)*, Rhodobacter capsulatus* (2) , *Rhodospirillum rubrum* (3), *Blastochloris viridis* (4) and *Rhodopseudomonas palustris* (5) with the *anf* genes labelled. Ticks are 1 kb apart. *anfHDKG* are the iron-only nitrogenase structural genes; *anfA* is regulatory, *anfOR* are of unknown function, and are also called *anf1* and *anf2* in *R. capsulatus*, *draT* is associated with post-translational regulation of the AnfH protein (6).


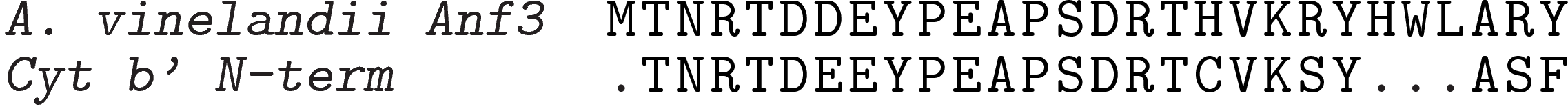


**Figure S2.** Alignment of the Anf3 sequence from *A. vinelandii* (Avin_49040) and the historical unpublished *Azotobacter vinelandii* cytochrome *b’* N-terminal sequence produced by Edman degradation (J. Schumacher, 1993, A Novel Cytochrome *b’* of *Azotobacter Vinelandii* and its Role in Nitrogen Fixation.” Diplomarbeit, University of Cologne), showing that these are the same protein. In the initial unpublished characterization of this protein the FAD was not identified in the spectrum and so the protein was classified as a cytochrome *b’*. The *A. vinelandii anf3* is predicted to be cotranscribed with *anfU* (Avin_49030), and is adjacent to the other *anf* genes encoding iron-only nitrogenase structural proteins and regulatory and assembly factors. AnfU is transcribed 5.7-fold more when the FeFe is expressed (7) and is predicted to be involved with assembly of the FeFe cluster, by analogy with NifU and MoFe (8).


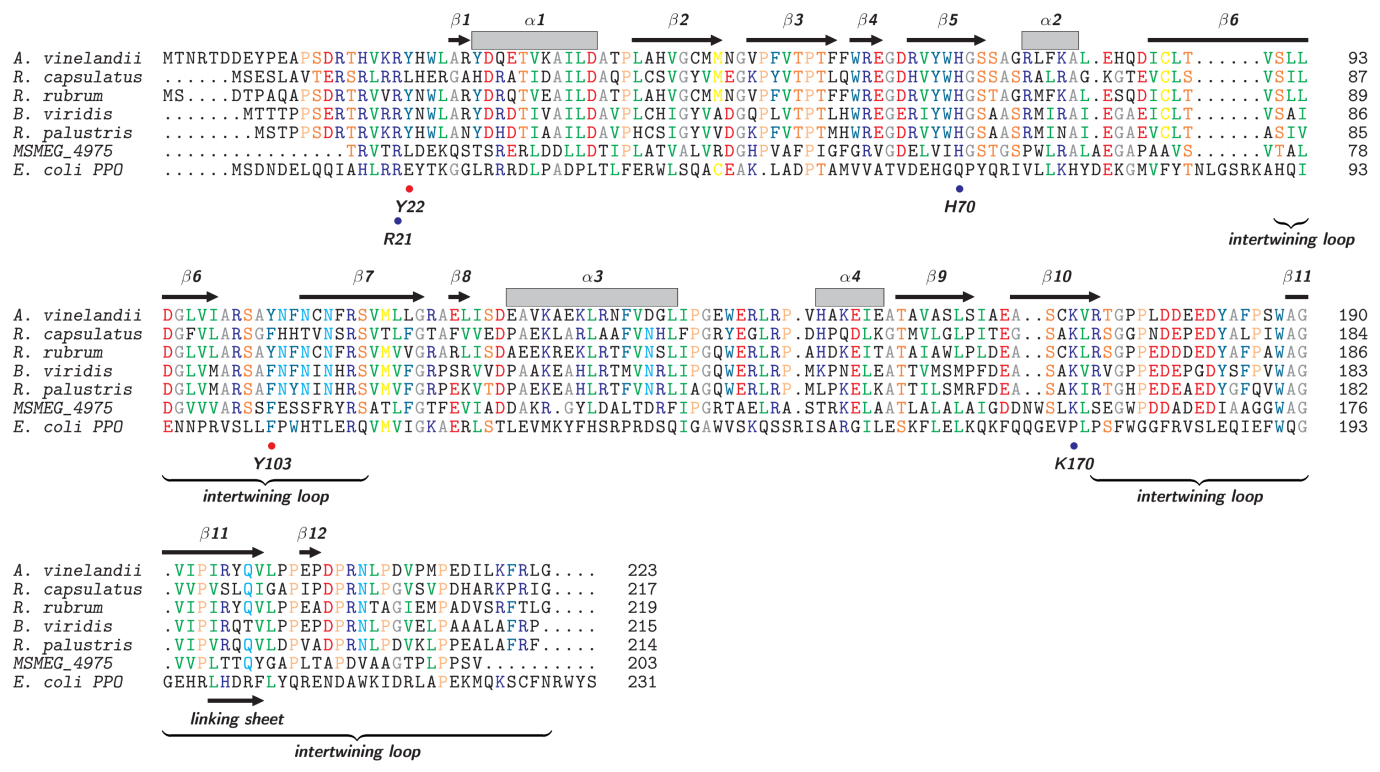


**Figure S3.** Alignment of the Anf3-like sequences from Fig S1, MSMEG_4975 and *E. coli* pyridoxine 5'-phosphate oxidase. The secondary structure of Anf3 of the *A. vinelandii* crystal structure is shown above the alignment. Residues mentioned in the text are also shown. The potential redox-active tyrosines 22 and 103 are not conserved among Anf3-like sequences. Alignment produced with ClustalX (9) and TeXshade (10).


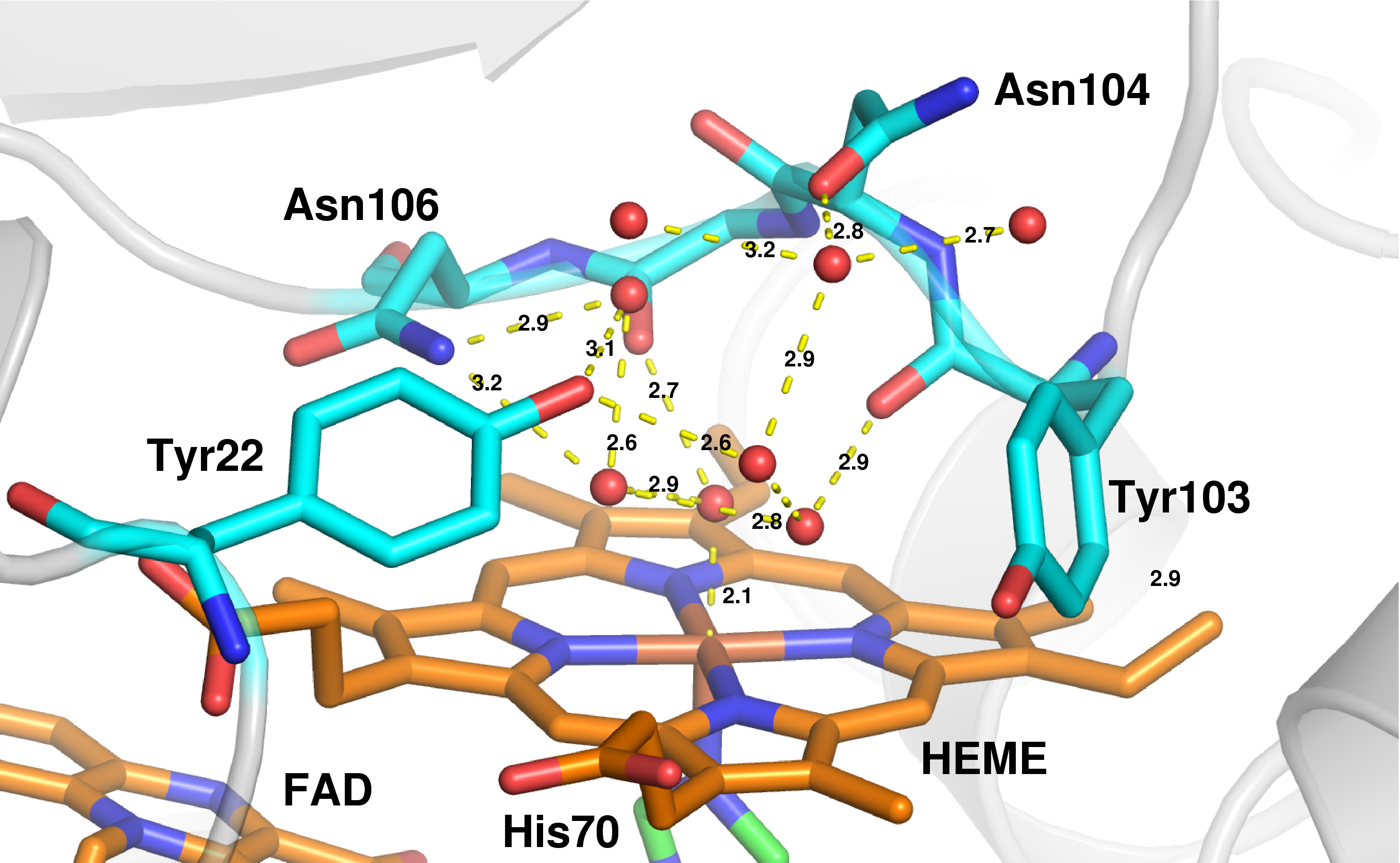


**Figure S4.** Structure of the Anf3 active site selected residues shown as sticks, with waters and hydrogen bonds shown with distances labelled in Ångström, as well as the heme iron to axial water. The view is from the outside of the protein.


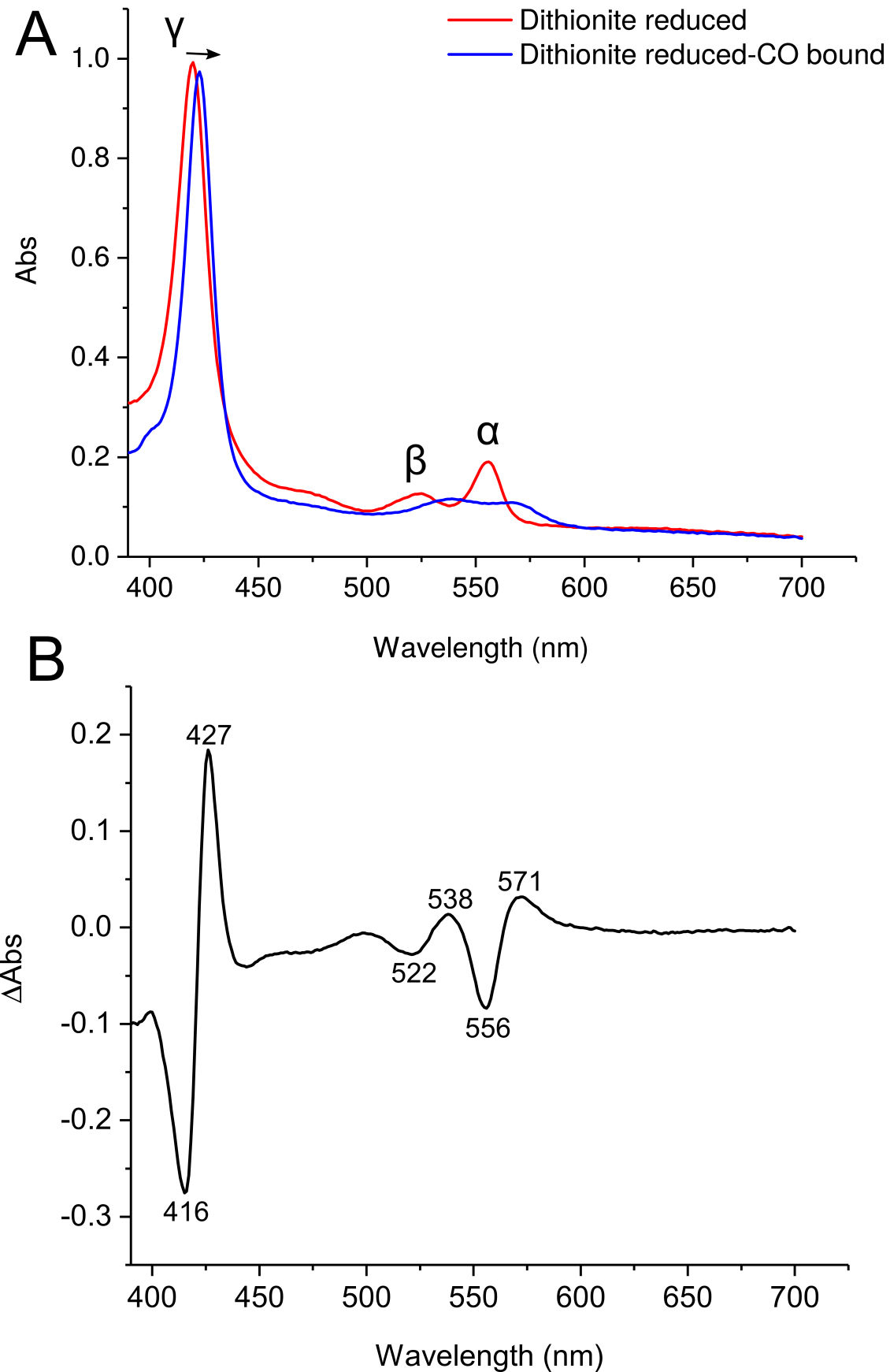


**Figure S5.** **Absorption spectra of reduced Anf3 and its ferrous-carbon monoxide complex. (**A) Absorption spectrum of reduced Anf3 (red) and reduced Anf3 carbon monoxide complex (blue). The Soret γ band shifts from 420 nm to 423 nm and the heme α (556 nm) and β (524 nm) bands disappear, which is indicative of a CO-bound *b*-type cytochrome. (B) Difference spectrum (CO-bound ‑ reduced) with troughs at 416 nm, 522 nm, and 556 nm and peaks at 427 nm, 538 nm and 571 nm. Dithionite-reduced Anf3 was sparged with CO gas for 5 minutes to generate the CO-bound complex.


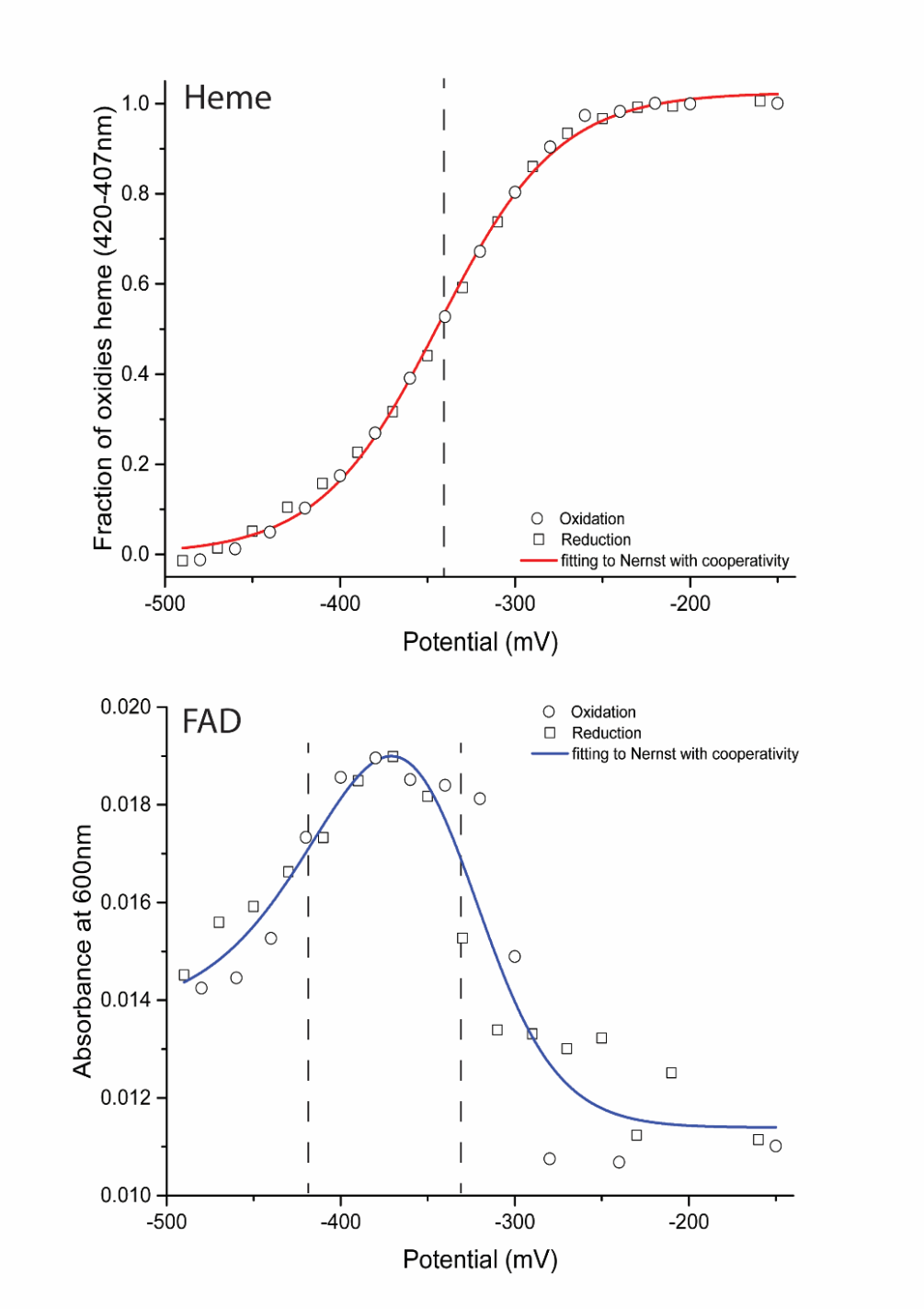


**Figure S6.** Fitting of heme and FAD reduction/oxidation data obtained using wavelengths where the spectroscopic interference of the other cofactor was minimised. Heme was followed at 420 nm with absorbance at 407 nm subtracted. At these wavelengths the extinction coefficient of the heme is 10 times higher than the FAD. The FAD was followed at 600 nm where only the FAD semiquinone (FAD_sq_) absorb and where the heme has negligible absorption. The data clearly show the 1 electron reduction of FAD to form the semiquinone followed by its 1 electron reduction to form the fully reduced FAD hydroquinone (FAD_hq_). The dashed lines represent the values of E_m_ obtained from the fittings of the data at 556 nm and 465 nm and show excellent agreement with these data.





**Figure S7.** Comparison of the fitting of the heme redox titration data using a Nernst equation with a cooperative parameter that takes into account the reciprocal interaction between the redox cofactors and a standard Nernst equation with n=1 electrons. At high potentials, where the cofactors are oxidised, it is easier to reduce the heme than expected from the ideal n=1 conditions — the data points sit below the n=1 curve, while at low potentials, where FAD_sq_ and some FAD_hq_ are present, it becomes harder to reduce the heme — the data points are now above the n=1 curve.


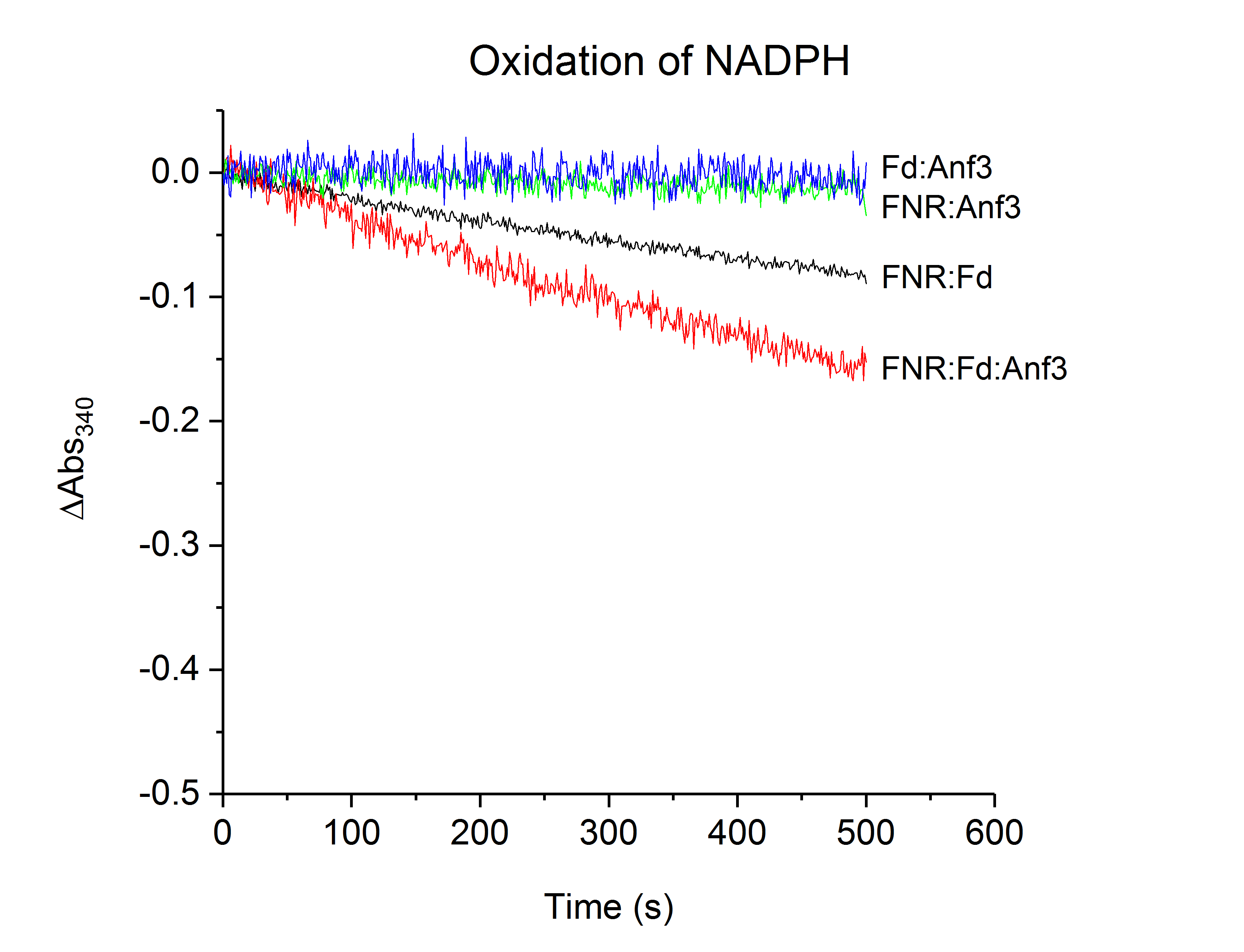


**Figure S8.** The kinetics of NADPH oxidation, measured by changes in absorbance at 340 nm. The addition of Anf3 increased the rate of NADPH oxidation by 2.3 fold

**Supplementary References**

1. Setubal, J., dos Santos, P., Goldman, B., Ertesvåg, H., Espin, G., Rubio, L., Valla, S., Almeida, N., Balasubramanian, D., Cromes, L., Curatti, L., Du, Z., Godsy, E., Goodner, B., Hellner-Burris, K., Hernandez, J., Houmiel, K., Imperial, J., Kennedy, C., Larson, T., Latreille, P., Ligon, L., Lu, J., Maerk, M., Miller, N., Norton, S., O’Carroll, I., Paulsen, I., Raulfs, E., Roemer, R., Rosser, J., Segura, D., Slater, S., Stricklin, S., Studholme, D., Sun, J., Viana, C., Wallin, E., Wang, B., Wheeler, C., Zhu, H., Dean, D., Dixon, R., and Wood, D. (2009) Genome sequence of *Azotobacter vinelandii*, an obligate aerobe specialized to support diverse anaerobic metabolic processes. *J. Bacteriol.* **191**, 4534–4545

2. Strnad, H., Lapidus, A., Paces, J., Ulbrich, P., Vlcek, C., Paces, V., and Haselkorn, R. (2010) Complete genome sequence of the photosynthetic purple nonsulfur bacterium *Rhodobacter capsulatus* SB 1003. *J. Bacteriol.* **192**, 3545–3546

3. Lonjers, Z., Dickson, E., Chu, T.-P., Kreutz, J., Neacsu, F., Anders, K., and Shepherd, J. (2012) Identification of a new gene required for the biosynthesis of rhodoquinone in *Rhodospirillum rubrum*. *J. Bacteriol.* **194**, 965–971

4. Liu, L.-N., Faulkner, M., Liu, X., Huang, F., Darby, A., and Hall, N. (2016) Revised Genome Sequence of the Purple Photosynthetic Bacterium Blastochloris viridis. *Genome Announc.*

5. Larimer, F., Chain, P., Hauser, L., Lamerdin, J., Malfatti, S., Do, L., Land, M., Pelletier, D., Beatty, T., Lang, A., Tabita, R., Gibson, J., Hanson, T., Bobst, C., Torres, J. T., Peres, C., Harrison, F., Gibson, J., and Harwood, C. (2004) Complete genome sequence of the metabolically versatile photosynthetic bacterium *Rhodopseudomonas palustris*. *Nat. Biotechnol.* **22**, 55–61

6. Masepohl, B., Krey, R., and Klipp, W. (1993) The draTG gene region of *Rhodobacter capsulatus* is required for post-translational regulation of both the molybdenum and the alternative nitrogenase. *J. Gen. Microbiol.* **139**, 2667–2675

7. Hamilton, T., Ludwig, M., Dixon, R., Boyd, E., Dos Santos, P., Setubal, J., Bryant, D., Dean, D., and Peters, J. (2011) Transcriptional profiling of nitrogen fixation in *Azotobacter vinelandii*. *J. Bacteriol.* **193**, 4477–4486

8. Zhao, D., Curatti, L., and Rubio, L. (2007) Evidence for *nifU* and *nifS* participation in the biosynthesis of the iron-molybdenum cofactor of nitrogenase. *J. Biol. Chem.* **282**, 37016–37025

9. Larkin, M. a, Blackshields, G., Brown, N. P., Chenna, R., McGettigan, P. a, McWilliam, H., Valentin, F., Wallace, I. M., Wilm, a, Lopez, R., Thompson, J. D., Gibson, T. J., and Higgins, D. G. (2007) Clustal W and Clustal X version 2.0. *Bioinformatics*. **23**, 2947–8

10. Beitz, E. (2000) TeXshade: shading and labeling of multiple sequence alignments using LaTeX2e. *Bioinformatics*. **16**, 135–139

11. Adams, P., Afonine, P., Bunkóczi, G., Chen, V., Davis, I., Echols, N., Headd, J., Hung, L., Kapral, G., Grosse Kunstleve, R., McCoy, A., Moriarty, N., Oeffner, R., Read, R., Richardson, D., Richardson, J., Terwilliger, T., and Zwart, P. (2010) *PHENIX*: a comprehensive Python-based system for macromolecular structure solution. *Acta Crystallogr. Sect. D*. **66**, 213–221
